# Interaction between Selected Adipokines and Musculoskeletal and Cardiovascular Systems: A Review of Current Knowledge

**DOI:** 10.3390/ijms242417287

**Published:** 2023-12-09

**Authors:** Olga Sierawska, Marek Sawczuk

**Affiliations:** 1 Institute of Physical Culture Sciences, University of Szczecin, 71-065 Szczecin, Poland; marek.sawczuk@usz.edu.pl; 2 Doctoral School, University of Szczecin, 70-384 Szczecin, Poland

**Keywords:** adipokines, adipose tissue, physical activity, adiponectin, leptin

## Abstract

Adipokines are substances secreted by adipose tissue that are receiving increasing attention. The approach to adipose tissue has changed in recent years, and it is no longer looked at as just a storage organ but its secretion and how it influences systems in the human body are also looked at. The role of adipokine seems crucial in developing future therapies for pathologies of selected systems. In this study, we look at selected adipokines, leptin, adiponectin, chemerin, resistin, omentin-1, nesfatin, irisin-1, visfatin, apelin, vaspin, heparin-binding EGF-like growth factor (HB-EGF), and TGF-β2, and how they affect systems in the human body related to physical activity such as the musculoskeletal and cardiovascular systems.

## 1. Introduction

Despite the old view that adipose tissue serves only as an energy storage and thermoregulation mechanism, it is now known to be a secretory organ. With the help of hormonal, paracrine, and autocrine signals, it regulates the body’s metabolism [1]. In humans, white adipose tissue (WAT) is found in the greatest amount, which is stored mainly under thin people’s skin (subcutaneous adipose tissue, SAT) and then in the visceral area (visceral adipose tissue, VAT). In addition to WAT, brown adipose tissue (BAT) is in the supraclavicular, paravertebral, and mediastinal regions. WAT, under the influence of cold exposure and high physical activity (PA), can acquire the characteristics of BAT, i.e., an increase in the expression of uncoupling protein-1 (UCP-1) and an increase in the number of mitochondria, in a process called WAT browning, resulting in the formation of beige adipocytes [2]. Primarily, the regulators secreted by adipose tissue are adipokines such as leptin, adiponectin, chemerin, resistin, omentin-1, nesfatin, irisin-1, visfatin, apelin, vaspin, heparin-binding EGF-like growth factor (HB-EGF), and TGF-β2 [1].

In addition to their effects on metabolic regulation, increasing attention is being paid to the systemic effects of adipokines on the body. These include the impact on whole systems, such as the muscular, skeletal, immune, and cardiovascular systems, and on processes like spermatogenesis, insulin resistance, and tumorigenesis [3,4].

In this work, we review the available literature on the effects of adipokines on systems in the human body, particularly those related to PA. Reviewing the available data will help determine whether knowledge of adipokines is sufficient to develop strategies to improve physical capacity in patients with various conditions. This is particularly important in rehabilitation after injury or musculoskeletal disease, and is also important for more effective therapies for overweight/obese patients. We searched PubMed and Scholar scientific databases for recent articles analysing the topic under study using keywords such as “adipose tissue cross-talk”, “[adipokine name] muscular system”, “[adipokine name] skeletal system”, “[adipokine name] cardiovascular”, and “[adipokine name] physical activity”. Specifically, reports from the last 5 years were considered, and available older data were necessary for a comprehensive view.

## 2. Musculoskeletal System

### 2.1. Muscular System

The muscular system is essential in maintaining the human body’s balance of glucose and fatty acids [5]. One of the main activities that engages the muscular system is PA. During exercise, circulating plasma glucose and muscle glycogen stores are used by skeletal muscles as a source of energy [6]. Intense exercise stimulates the ability of muscle tissue to oxidise lipids in both normal-weight and overweight/obese individuals [7]. Training improves glucose uptake and adrenergic receptor sensitivity to catecholamines in adipose tissue and lipoprotein lipase (LPL) activity [6].

**Figure 1:**
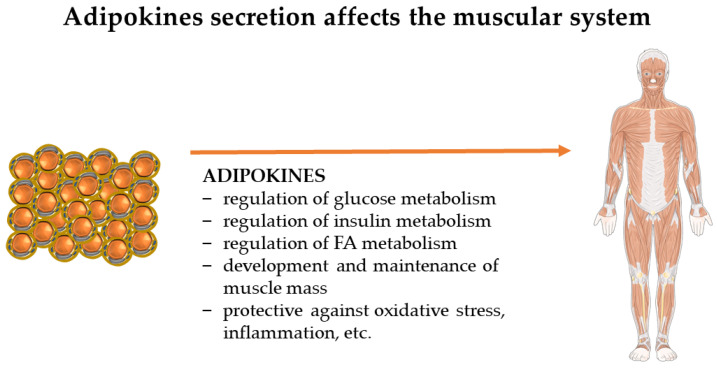
Adipokines secreted by adipose tissue have an impact on the entire human muscular system. The figure shows the essential effects exerted by leptin, adiponectin, chemerin, resistin, omentin-1, nesfatin, irisin-1, visfatin, apelin, vaspin, HB-EGF, and TGF-β2. The figure was partly generated using Servier Medical Art, provided by Servier, licensed under a Creative Commons Attribution 3.0 license. (FAs—fatty acids.)

During PA, glucose stores and stored fat are used up first [6]. The proper ratio between fat and muscle tissue is key to proper insulin regulation. Insulin receptors are found in both tissues and are structurally identical, but there is a difference between their actions. It is more beneficial for glucose clearance when the muscle insulin receptor is activated [8]. However, when there is a change in the ratio of fat and muscle levels, there are problems in the regulation of glucose metabolism. Although some insulin is produced, it is insufficient, leading to increased production by the pancreas [8]. Insulin resistance manifests itself through the inadequate ability of insulin to reuptake glucose in the tissues of skeletal muscle, adipose tissue, and the liver [6]. In addition to regulating glucose and insulin metabolism, adipokines affect other elements in the muscle system. The most common ones are shown in Figure 1, and the rest are described in detail below.

Leptin is one of the most important and well-studied adipokines. It regulates the hypothalamic satiety centre and body mass; its levels also reflect an individual’s energy stores and nutritional status [9]. Among people with excess body fat, leptin levels are higher than those with normal body fat [10]. However, elevated leptin levels affect factors involved in feelings of satiety and appetite suppression, such as the cocaine and amphetamine-regulated transcript (CART) system and neuropeptide Y [2]. In the pathology and physiology of the muscular system, leptin plays a significant role. It is involved in the oxidation and absorption of glucose and free fatty acids (FFAs) [11,12]. Leptin inhibits lipogenic pathways in skeletal muscle [13]. Regulating insulin growth factor-binding protein-2 sensitises skeletal muscle to insulin [13]. In addition, leptin has been shown to have roles in regulating capillarisation and skeletal muscle angiogenesis [13,14]. It acts as a local paracrine signalling molecule [13]. Leptin receptors have been identified in skeletal muscle, and this has been confirmed by the discovery of a 170-kDa OB-R isoform present in muscle fibres but absent in fat tissue [15,16]. PA increasing muscle strength affects lower leptin levels in obese children and overweight/obese middle-aged patients (>45 yrs.) [15,17]. Specifically, leptin production is impaired by promoting muscle mass gain or resistance or weight training. It is thought that this may be related to the increased expression of leptin receptors present in muscle [17]. Men typically have higher muscle mass than women and have lower leptin levels [17]. Patients with increased leptin levels, such as middle-aged women, seniors, patients with knee osteoarthritis, and adults with chronic Huntington’s disease, are associated with PA disorders and poorer muscle quality [14].

Adiponectin, like leptin, is one of the most important and well-studied adipokines. It is a regulator with a wide range of functions—it has both pro- and anti-inflammatory effects [18] and is secreted by adipocytes from WAT and BAT [12]. Adiponectin levels are reduced in people with excess visceral adipose tissue compared to those with normal adipose tissue levels [10]. Adiponectin comes in two forms: low-molecular-weight with anti-inflammatory effects and high-molecular-weight with pro-inflammatory effects [19]. Adiponectin is also involved in pathologies and physiologies of the muscular system, where it is expressed [11,20]. Its main role in skeletal muscle is to down-regulate glucose production and up-regulate muscle insulin sensitivity, energy expenditure, and fatty acid oxidation by regulating 5′AMP-activated protein kinase (AMPK) activation in muscle cells [10,15,21]. The regulation of insulin sensitivity by adiponectin in skeletal muscle occurs through (a) the activation of AMPK pathway signalling and (b) the activation of oxidation by PGC-1α [21,22]. Additionally, it regulates muscle contraction strength by controlling calcium concentration, governs the development and maintenance of muscle mass, has a protective effect in states of muscle atrophy, is associated with oxidative pathways and energy metabolism, regulates inflammation and oxidative stress by acting as a regulator of the M1 and M2 macrophage phenotype, stimulates autophagy, and has a neuroprotective effect during exercise [20,21,23].

Stimulating the AdipoR-1 receptor (enriched in skeletal muscle) can act as an autocrine or paracrine. In a series of animal experiments, increased adiponectin levels were observed during weeks of moderate physical activity but not in all [20,21]. In humans, these results remain contradictory. One series of resistance or aerobic exercise does not affect changes in adiponectin levels. PA series (weekly or monthly) keep its levels unchanged or increased [20].

Resistin (also described as adipocyte-specific secretory factor, ADSF, and found in inflammatory zone 3, FIZZ3) is secreted by macrophages and infiltrating monocytes in white adipose tissue [12]. It regulates the production of pro-inflammatory cytokines and has a role in energy homeostasis [24]. Resistin, in turn, impairs the AMPK pathway, thereby negatively affecting glucose homeostasis in muscles [25]. Also, it affects lipid deposition in skeletal muscle, destabilises myogenesis, alters metabolism in myotubes, and reduces the ability of muscles to absorb fatty acids [22].

The main form of circulating omentin is omentin-1, otherwise known as interlectin-1 [24]. It is produced in visceral adipose tissue and expressed in vascular cells [26]. Data regarding omentin-1 are inconsistent. Some sources report its sensitivity to a specific threshold of PA intervention and others report that changes are due to overall improvements in body composition resulting from the implementation of PA or dietary intervention. Studies show an increase/no increase after training in different types of patients studied, but with a particular focus on patients with obesity or diabetes [27,28]. Further research is needed to determine its role in the musculoskeletal system.

Chemerin is encoded by the retinoic acid receptor responder 2 gene (RARRES2) or tazarotene-induced gene 2 (*TIG2*). It is highly expressed in white adipose tissue (WAT), the liver, lung, intestine, and kidney [24]. Chemerin appears to be one of the adipokines most associated with the musculoskeletal system. Chemerin down-regulates insulin signalling, induces insulin resistance, inhibits insulin receptor substrate 1 and glycogen synthase kinase phosphorylation, and impairs glucose uptake in skeletal muscle [29,30,31]. The effects of chemerin on these processes probably occur through the activation of MAP kinase p38, extracellular signal-regulated kinase (ERK)-1/2, p56, and nuclear factor kappa B (NFkB) [29,30,32,33]. Chemerin also increases the pro-inflammatory and insulin-influencing cytokines interleukin-6 (IL-6) and TNF-α [31]. Increased chemerin levels have been observed in people with metabolic syndrome [32]. Chemerin also affects the mitochondria in muscle cells, which are also involved in regulating insulin resistance, reducing their content, and increasing the production of mitochondrial ROS [31].

Nesfatin is a peptide consisting of 82 amino acids that affect lipid metabolism, the cardiovascular system, emotions, and reproduction, as is essential for inducing a feeling of satiety [24,34]. There are various data on the involvement of nesfatin-1 in PA-related processes, where a decrease or increase in its concentration after PA is indicated. It is noted that its levels are related to cortisol and adrenocorticotropin levels, which increase in response to eccentric resistance training, suggesting its role in stress as well. In addition, nesfatin-1 regulates energy and affects insulin sensitivity [35,36]. Using processes in muscle such as increasing insulin-stimulated AKT activity, glucose aspiration, p-AMPK and acetyl-CoA carboxylase expression, and stimulating GLUT4 translocation of the membrane lowers blood glucose levels and reduces glucose and insulin resistance [37]. These processes also take part in the oxidation of fatty acids and their normalisation in muscles [37,38].

Irisin (FNDC5) is a peptide that is 112 amino acids long and an important chemical messenger involved in metabolism [39]. The surge in irisin-1 levels associated with PA makes one wonder whether it lies on the borderline of being an adipokine versus a myokine [27,28,40]. PA activates increased levels of AMPK, which leads to PGC-1α phosphorylation. Then, PGC-1α up-regulates the secretion of FNDC5, irisin precursor, and irisin alone. It also activates PPARγ, which up-regulates the secretion of FNDC5 and irisin [35]. It is involved in maintaining glucose homeostasis, reducing lipid accumulation in muscle, and developing and regenerating muscle primarily in response to damage from contractile activity and oxidative stress [40,41,42]. The higher the patient’s muscle mass, the higher the concentration of circulating irisin-1 [40]. Since it can cross the blood–brain barrier, the effects of this PA-induced adipokine on the brain have been looked at and found to improve both learning and memory and have a protective effect [41].

Visfatin is mainly synthesised by visceral adipose tissue, and its synthesis occurs in macrophages residing and infiltrating this tissue and in subcutaneous adipose tissue [12,24,26]. Visfatin in myocytes binds and activates the insulin receptor, which causes an effect that only mimics the action of insulin, as confirmed by studies showing the lack of involvement of insulin signalling pathways in the activity of visfatin [43]. It can affect glucose and fatty acid metabolism in skeletal muscle, and although it stimulates glucose transport, the effect of visfatin on fatty acid metabolism still needs further study [5]. Also, it can alter the contractile properties of muscles by increasing the expression of MHC I, MHC IIa, and MHC IIb [44]. However, visfatin levels in muscles do not seem to be increased by PA, the opposite of what happens in AT, where its levels increase after PA [45].

Apelin is defined as a regulatory peptide, which in humans occurs in active forms, apelin-36, apelin-17, apelin-13, and the pyroglutamate form of apelin-13, and is involved in the processes of maintaining the body’s homeostasis [46]. In skeletal muscle, apelin regulates glucose and FA metabolism, increasing insulin sensitivity by activating paracrine or autocrine. Increased Akt phosphorylation in muscle, through AMPK activation, increases both FA oxidation in muscle and muscle capillarisation, improving muscle metabolism and function [47,48,49]. The regulation of glucose metabolism occurs through the AMPK pathway [47]. Animal studies have shown the effect of apelin on mitochondria biogenesis in muscle [47,50]. This can also be considered a myokine because of its active response to PA [49]. 

Vaspin belongs to the serpin family and is produced mainly in visceral adipose tissue. Circumdian rhythms regulate its secretion, occurring after meals [26,51]. Vaspin has higher expression in the muscles of obese people than in healthy people. In myotubes of obese individuals, it induces activation of the PI3K-AKT signalling pathway, thereby increasing insulin sensitivity [52]. As muscle mass increases, the vaspin level rises [53]. It can also act on the cell surface as a receptor [54]. 

Heparin-binding growth factor-like EGF (HB-EGF) is a member of the epidermal growth factor (EGF) family and a potent growth factor for vascular smooth muscle cells [55]. HB-EGF increases insulin sensitivity and regulates glucose uptake in skeletal muscle [56]. It is also involved in smooth muscle hyperplasia [57].

TGF-β2 is an adipokine with developmental regulatory implications that has recently been reported to play roles in physical activity [58]. It increases TGF-β2 expression, resulting in increased insulin sensitivity, and improves glucose tolerance and FA oxidation, with a pronounced and significant involvement of lactate [58]. Also, TGF-β2 inhibits myoblast differentiation [59]. Elevated TGF-β2 levels occur in Duchenne muscular dystrophy patients (DMD) [60].

### 2.2. Skeletal System

**Figure 2:**
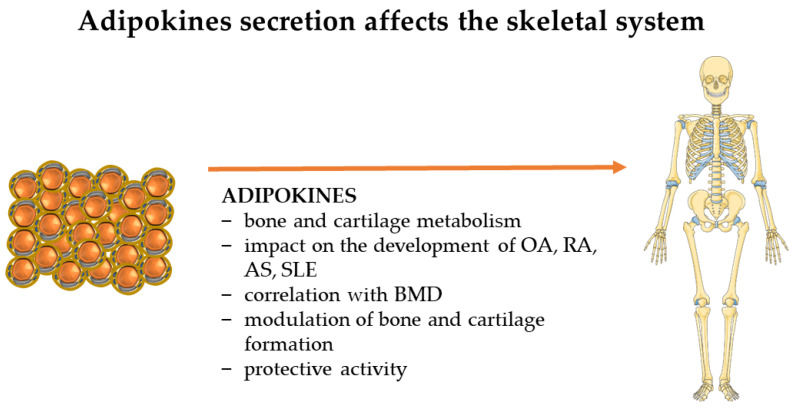
Adipokines secreted by adipose tissue have an impact on the entire human skeletal system. The figure shows the most important effects exerted by leptin, adiponectin, chemerin, resistin, omentin-1, nesfatin, irisin-1, visfatin, apelin, vaspin, HB-EGF, and TGF-β2. The figure was partly generated using Servier Medical Art, provided by Servier, licensed under a Creative Commons Attribution 3.0 unported license. (OA—osteoarthritis; RA—rheumatoid arthritis; AS—spondylitis; SLE—systemic lupus erythematosus; BMD—bone mineral density.)

PA is heavily involved in the molecular workings of the skeletal system. It has a positive effect on bone mineral density (BMD) and bone mineral content (BMC), increases bone mass, and, most importantly, has been shown to play a vital role in the prevention of osteoporosis and in reducing brittleness [61,62]. The role of PA in postmenopausal women is often highlighted, as they tend to have lower PA levels, with an associated loss of BMD [63]. PA is essential in every stage of life, not only in older people, but especially in children, whose skeletons are growing [61]. Also, it has a crucial role during puberty, where the impact on BMC growth is greater than after puberty [64]. Introducing weight-bearing exercises in obese children positively affects bone health, strength, and bone mass [65]. PA should be regular and >240 min/week, as below this value, no significant changes have been observed in patients [61]. Also, bone metabolism and other effects are regulated by adipokines, whose levels increase or decrease in response to exercise [66], and the main effects of adipokines on the skeletal system are shown in Figure 2. 

Leptin’s ability to regulate the expression of the receptor activator of nuclear factor KΒ ligand (RANKL) and neuropeptides in the hypothalamus and induce activation of the sympathetic nervous system gives it the ability to modulate bone formation [3,67]. The ratio of RANKL to osteoprotegerin (OPG), a substance secreted by osteoblasts, is important in bone remodelling [20]. High leptin levels are associated with a low risk of bone fractures [68]. Serum leptin levels are positively correlated with whole-body BMD, especially in women, in the lumbar spine, femoral neck, total hip, and radius bone [3,69]. Patients with adolescent idiopathic scoliosis (AIS), a complex spinal deformation, have lower leptin levels than healthy people [67]. Leptin receptors are also expressed in cartilage cells [3], and so they are also involved in cartilage metabolism, where their levels are elevated when there is a high degree of cartilage damage in a patient [70]. Hence, patients with osteoarthritis (OA) have elevated levels of it, and leptin itself has been found to have an impact on the development of the disease [70]. In addition to OA, leptin is reported to influence the pathogenesis of diseases such as rheumatoid arthritis (RA), ankylosing spondylitis (AS), systemic lupus erythematosus (SLE), or heterotopic ossifications of the posterior longitudinal ligaments and spine [19]. 

Adiponectin decreases osteoclast activity. Furthermore, activating RANKL and inhibiting OPG production increases osteoblast differentiation, and these processes positively affect bone homeostasis [24,71]. The correlation of adiponectin levels with whole-body BMD is still a debatable topic, as some studies show a negative correlation with BMD in the lumbar spine, the entire hip in men and postmenopausal women, and the femoral neck in premenopausal women [68], and others do not support this thesis [3]. However, studies with a group of men have indicated that regardless of body composition and BMD, the risk of bone fractures increases as adiponectin levels in the blood rise [24]. Patients with OA have higher levels of adiponectin expression than healthy controls. However, whether it has a pathogenic or protective function in the disease and its recognition as a biomarker of OA progression remain debatable [72,73]. Elevated adiponectin levels are also seen in RA, psoriatic arthritis, and SLE [19], whereas reduced adiponectin levels are seen in women with osteoporosis [74]. Adiponectin receptors are expressed on osteoblasts and osteoclasts [3].

Resistin does not play an important role in regulating bone metabolism, and there is no significant correlation between it and BMD [24,68]. However, it may affect RA patients’ disease activity and joint damage [19]. Resistin levels were significantly higher in AS patients than healthy controls, indicating its pro-inflammatory function in developing the disease [19,75].

Data from preclinical and clinical studies on omentin-1 are so inconsistent that it is challenging to conclude its role in the pathogenesis of bone lesions [24]. It is known that omentin-1 is involved in bone metabolism [24]. However, it is still difficult to determine its exact role. On the one hand, omentin-1 has been observed to disrupt the balance of the RANKL/OPG ratio, which is important in bone remodelling [76]. On the other hand, the PI3K/Akt pathway promotes osteoblast proliferation, an important regulator in bone remodelling [77]. Omentin-1 has a negative correlation with BMD in premenopausal women and may inhibit bone formation in premenopausal women [76]. In patients with multiple sclerosis, omentin-1 was associated with higher BMD in the femoral neck [71]. Its increased levels may be a protective adaptation to osteopenia [71]. 

In vitro studies indicate the role of chemerin and its receptor CMKLR1 in the osteoblastogenesis of bone mineralisation and inhibition of osteoclastogenesis, making it negatively related to bone metabolism [78]. In addition to its effect on the bone remodelling cycle, it is negatively correlated with total BMD [24]. It is reported to be positively associated with the development of osteoporosis, and in postmenopausal osteoporotic patients its levels are lower than in healthy people [78,79].

There are still few studies on the effects of nesfatin-1 on the skeletal system in humans, and animal studies have shown its protective properties in bone metabolism, including positive effects on limiting bone mass loss, protecting against architectural changes, and increasing bone strength [24,80]. 

Irisin-1 is involved in reducing osteoclast differentiation and inhibiting osteoclastogenesis [81,82]. Also, it increases anabolic factors such as β-catenin, which induces osteoblast differentiation [82]. Irsin-2 has an inhibitory effect on the development of osteoporosis by increasing the number and activity of osteoblasts and suppressing sclerostin (Sost), which has an inhibitory effect on the formation of new bone [82]. Middle-aged and elderly patients with osteoporosis have lower irisin-1 levels and are positively correlated with BMD [81]. Increased irisin release during PA is one of the mechanisms involved in improving bone health [65].

In the case of visfatin, in vitro data indicate its roles in bone metabolism, but few in vivo data indicate such properties [24]. Additionally, attention is paid to its roles in the pathology and physiology of the musculoskeletal system [11]. There were no statistical correlations between visfatin levels and BMD [71,83]; the only correlation was across the hip joint [68]. RA patients had significantly higher levels of visfatin than healthy patients, hence the attention to its important pro-inflammatory role in RA pathogenesis and the possibility of using it as a potential therapeutic target [84]. 

By increasing osteoblast proliferation, enhancing osteoblastogenesis, and inhibiting their apoptosis, apelin plays a protective role for bone [24,85]. Apelin is involved in the regulation of skeletal homeostasis and the pathogenesis of OA [24].

Vaspin, on the other hand, increases bone formation, reduces resorption, and exhibits activity that modulates bone cell function, where it has a positive effect on osteoblasts and an inhibitory effect on osteoclasts [86]. In vitro studies indicate its roles in protecting osteoblasts from apoptosis and the modulating effect of vaspin on osteogenic differentiation [87]. Vaspin also shows a positive correlation with BMD, and its elevated levels occur in patients with RA [24,87]. 

HB-EGF influences bone formation and resorption by promoting osteoblast and chondrocyte proliferation. It also regulates the proliferation and differentiation of osteoblasts, chondrocytes, and bone marrow stem cell (BMSC) cultures [88]. 

TGF-β2 is an osteoinductive factor because it promotes bone and cartilage formation [89]. In addition, it impacts the bone repair process, including accelerating fracture healing, where its local injection promotes the healing of bone fractures [90].

## 3. Cardiovascular System

**Figure 3:**
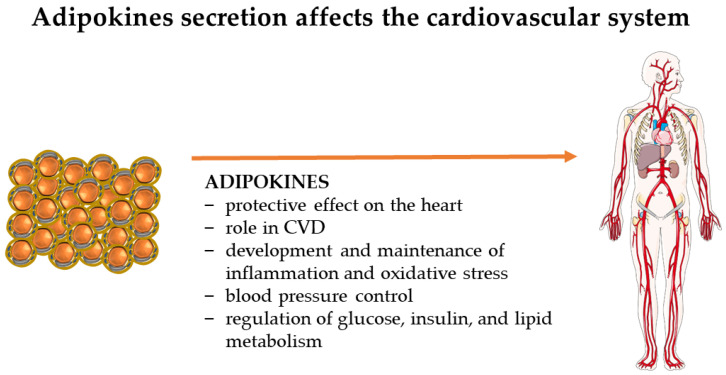
Adipokines secreted by adipose tissue impact the entire human cardiovascular system. The figure shows the essential effects exerted by leptin, adiponectin, chemerin, resistin, omentin-1, nesfatin, irisin-1, visfatin, apelin, vaspin, HB-EGF, and TGF-β2. The figure was partly generated using Servier Medical Art, provided by Servier, licensed under a Creative Commons Attribution 3.0 unported license. (CVD—cardiovascular disease.)

PA is associated with reduced risk of the development of cardiovascular disease (CVD) and CVD mortality [91]. Essential features of PA for the cardiovascular system are its positive effect on the homeostasis of oxidative metabolism, its protective role on myeloma, its impact on remodelling and improvements in cardiac performance, and its effect on increasing HDL and decreasing LDL in plasma, lowering systolic blood pressure and glycosylated haemoglobin levels, and regulating cytokine secretion [92,93]. PA is recognised as one of the effective treatments for CVD, but patients with the disease have lower levels of PA than those without CVD [94]. For PA to affect CVD, it is necessary to do it in moderate form for a minimum of 150 min/week or large form for a minimum of 75 min/week [63,95]. Notably, the abrupt cessation of PA for two weeks affects the loss of its positive effects on the cardiovascular system, as well as the reduced uptake of glucose in the muscles and, thus, the development of insulin resistance in them [63]. However, there is a flip side: long-time athletes have an increased risk of CVD, and it is noted that they have increased coronary calcium levels [95]. The evaluation of adipokines’ effects is essential for understanding the impact of PA on the cardiovascular system. The main effects of adipokines on the cardiovascular system are shown in Figure 3.

Leptin is an angiogenesis stimulant that has a protective or detrimental effect on the heart, depending on its concentration [26]. Decreased levels of leptin affect the homeostasis of cardiac metabolism, whereas its increased levels affect the development of systemic inflammation and increase the risk of CVD. The disease is the result of the increase in the number of constricted coronary arteries, increased blood pressure, and heart rate [96,97]. Animal studies have shown the beneficial effects of leptin on the heart, where animals with deletion of the leptin Lepr receptor in cardiomyocytes had impaired cardiac regeneration after myocardial infarction, thinner ventricular walls, reduced ejection fraction, and impaired glucose regulation [98]. Leptin reduces oxidative stress, improves mitochondrial function, and thus has an anti-apoptotic effect on cardiomyocytes [97]. Moreover, leptin also affects blood pressure control by reducing passive wall tension and vasoconstriction [99]. In addition, it has antihypertensive effects, regulates cardiac contractile function, and inhibits vascular smooth muscle cell (VSMC) proliferation in the aorta [97,98,99].

Adiponectin has anti-apoptotic, anti-inflammatory, anti-swelling, and antioxidant effects [100]. Adiponectin has a cardioprotective effect by affecting autophagy with hypertrophy, inhibiting atherogenesis and apoptosis, reducing lipotoxic damage and ROS production, and promoting cell viability [100,101,102]. Anti-inflammatory effects of adiponectin include its action on macrophages and endothelial cells [101]. It also has a blood-pressure-lowering effect [101]. Adiponectin is referred to as a “rescue hormone” because of its role in preventing heart injury [100]. The role of adiponectin in the regulation of glucose levels and insulin responsiveness is worth noting [100]. Adiponectin can prevent the development of atherosclerosis by regulating lipid metabolism and affecting NO and ROS production, reducing the effects of CVD by regulating the balance of glucose and lipid metabolism, and inhibiting inflammation and oxidative stress [26,96,100,101]. However, elevated levels of adiponectin are associated with the development of early pulp lesions in the carotid arteries and advanced CVD if their levels are elevated [102,103]. 

By regulating glucose and lipid levels, chemerin is involved in lipid deposition in endothelial cells and the progression of arteriosclerosis [104]. However, it can affect the progression of myiasis and other CVDs by stimulating adipogenesis, inflammation, and contraction and affecting thermogenesis, steroidogenesis, and insulin signalling [105]. Chemerin’s action as a chemoattractant affects the expression of inflammatory factors (e.g., IL-6, TNFα, and CRP), leading to abnormal endothelial secretion, inflammation of blood vessel walls, and increased adhesion of monocytes to endothelial cells [104]. Elevated levels of chemerin, as is the case in people with obesity, have an impact on kidney dyscrasia by acting on blood vessels, including increased production of ROS [106]. 

Resistin has detrimental effects on the heart [96]. This was indicated, among other things, using a humanised mouse model, where it acted immunoregulatory [107]. When circulating resistin was lowered in mice, improved cardiac function, reduced cardiac fibrosis, and apoptosis were observed [108]. Resistin is a substance that stimulates angiogenesis [26]. Resistin acts on endothelial dysfunction and CVD, where, for example, in patients with severe ischemic heart disease or hypertension, its levels are higher than in healthy controls, and the more influential the ischemic heart disease is that is diagnosed, the higher the serum resistin levels are [109,110,111]. Due to its negative impact, it is suggested as a biomarker of overall mortality [112]. 

The cardiovascular effects of omentin-1 have been described as antiatherosclerosis and protective, including protection against endothelial dysfunction and ROS-induced apoptosis, and it may also protect against FFA-induced cell proliferation and migration [12,96,113]. Reduced levels of omentin-1 are associated with poor prognosis in CVD and endothelial dysfunction in overweight patients, and elevated levels of omentin-1 are associated with improved endothelial function in patients with T2D and with increased insulin sensitivity and reduced BMI, blood pressure, and IL-6 and CRP-1 levels in patients without T2D [113].

Nesfatin affects the regulation of cardiovascular homeostasis [114] and has paracrine/autocrine effects on the heart. It also participates in the regulation of glucose metabolism in cardiomyocytes by affecting GLUT-4 [115]. Nesfatin regulates blood pressure by acting centrally and peripherally. It is assumed that stimulating the secretion of oxytocin activates the melanocortin glia [114,115,116]. Increased nesfatin levels in patients may be a biomarker for the risk of developing hypertension [117]. Furthermore, nesfatin has a protective effect on the heart by inhibiting the mechanisms of heart failure pathogenesis, including a reduction in the impact of infarction in ischemic or reperfusion injury, improving recovery from ischemic contraction, and reducing levels of cardiac troponin-T and inflammation, apoptosis, and necrosis [114,115]. 

Irisin levels are indicated as a biomarker of CVD progression, including acute myocardial infarction (MI) and coronary artery disease (CAD) [118]. Studies also show correlations of its levels with the onset of hypertension in patients. However, the results are inconsistent regarding whether the effect relates to elevated or reduced adipokine levels. It has been noted that decreased irisin levels in patients with secondary hypertension may be associated with the development of inflammation [118]. Irisin affects blood pressure control by regulating blood vessels, where it can constrict or relax them. In patients after a myocardial infarction, irisin levels are elevated, which negatively affects the development of cardiovascular events and heart cell damage. However, its high levels may affect the repair of cardiac muscle cells, and the exact mechanism of this process still needs to be investigated. Irisin inhibits oxidative stress and attenuates Akt signalling activation, improving cardiac remodelling. Its cardioprotective effects also include reducing oxidative stress damage and apoptosis. Furthermore, during acute hypoxemia, irisin has a protective effect [40,119]. Attention is also drawn to its roles in atherosclerosis, where it has a protective effect. Patients with reduced serum irisin levels are at risk of developing the disease [40]. 

On the one hand, visfatin has a protective effect on the heart, including during ischemia, stimulates angiogenesis, and has proliferative effects, including the proliferation of cardiac fibroblasts, and pro-inflammatory effects [26,120]. However, on the other hand, its influence on CVD progression has been documented: in atherosclerosis, visfatin is responsible for endothelial dysfunction; in patients with ischemic heart disease and acute myocardial infarction (AMI), its levels are associated with myocardial changes; and in myocardial fibrosis, its proliferative properties affect its development [120]. 

In the cardiovascular system, apelin has a protective function: it controls blood pressure, maintains the homeostasis of body fluids, and improves carbohydrate and fat metabolism [121,122]. Depending on the concentration, it has a protective or harmful effect on the heart [96]. In addition, it affects vasodilation, regulates myocardial contractility, angiogenesis, and energy metabolism, and has pro-relaxant, inotropic, anticoagulant, antioxidant, and anti-inflammatory effects [121,122]. 

Vaspin has a protective effect on the vasculature by protecting against FA-induced apoptosis and inhibiting vascular cell proliferation and migration [12]. Also, vaspin has a protective effect on endothelial progenitor cells [24,123]. The most considerable debate continues to be about the role of vaspin in atherosclerosis, as it has been reported to have anti-atherosclerotic properties by modulating inflammation and also play a role in the pathophysiology of atherosclerosis by engaging in the pro-inflammatory pathway of atherosclerosis development [123,124]. Its anti-inflammatory action acts in vascular smooth muscle cells and may attenuate myocardial remodelling and improve cardiovascular prognosis in patients with AMI [124]. Patients with CAD have lower levels of vaspin than healthy controls [125]. 

HB-EGF has been identified as a critical factor for maintaining cardiac function [126]. Through the activation of EGFR, HB-EGF affects normal valvulogenesis [127]. The roles of HB-EGF in the pathophysiology of myiasis are also indicated [128]. 

TGF-β2 influences the stability of the pulp plaque by regulating inflammation and matrix degradation, indicating its protective role in the pulp process [129]. The functions of TGF-β2 in promoting cardiac myogenesis and the induction of epithelial–mesenchymal transition are also indicated [130].

## 4. Conclusions

Adequate body scale management and maintaining the right muscle and fat tissue ratio are essential for maintaining health [8]. As humans develop more and more adipose tissue, it is necessary to look at its mechanisms, especially its ability to communicate throughout the body. As outlined, the impact of substances secreted by adipose tissue is significant. Physical activity and a proper diet help to maintain good health, which influences the balance of adipokine secretion by adipose tissue, the opposite of what happens in states of obesity and metabolic inflammatory diseases [131]. It is essential to pay attention to and develop a new view of adipokines as factors that regulate several processes in the body, including energy expenditure, appetite and satiety, inflammation or glucose metabolism, and insulin sensitivity [132]. PA as a factor that regulates adipokine levels in the blood has great potential, especially with the known role of PA in reducing inflammation and the dysregulation of adipokine secretion that occurs with it, including adiponectin, visfatin, omentin-1, or leptin [133]. 

This study’s main limitation is the incomplete picture of the effects of adipokines on humans, which is due to the fact that adipokines are currently an area of intensive research. As we have presented in this work, not all adipokines have been thoroughly studied for their effects on systems in the human body, and some data are contradictory and, therefore, need to be extensively clarified. More research is needed on the new topic of substances secreted by adipose tissue in health and disease states and in physically active and non-active individuals. For this reason, it is also challenging to propose a practical use of the collected knowledge in sports medicine. Only a thorough understanding of the secretory mechanisms of adipose tissue will make it possible to determine whether adipokines can be therapeutic targets in diseases related to the musculoskeletal and cardiovascular systems. 

However, gathering knowledge about adipokines deepens the understanding of the mechanisms through which adipokines affect the musculoskeletal and cardiovascular systems. This expanded understanding will broaden knowledge of the role of these substances in bodily functions. Identifying the potential benefits and risks associated with regulating adipokine secretion in the body during PA will be necessary for developing treatment and prevention strategies for diseases related to the cardiovascular and musculoskeletal systems.

## Data Availability

No new data were created or analyzed in this study. Data sharing is not applicable to this article.
